# The International Reach of the *International Journal of Women's Dermatology* and the Women's Dermatologic Society

**DOI:** 10.1016/j.ijwd.2021.05.004

**Published:** 2021-05-21

**Authors:** Dedee F. Murrell, Jenny E. Murase

**Affiliations:** a The George Institute of Global Health, Sydney, Australia; b Sydney Faculty of Medicine, University of New South Wales, Sydney, Australia; c Department of Dermatology, University of California, San Francisco, California; d Department of Dermatology, Palo Alto Foundation Medical Group, Mountain View, California

When the board of the Women's Dermatologic Society (WDS) voted in 2013 to approve the proposal by board members Drs. Dedee Murrell and Jane Grant-Kels to explore the feasibility of developing the *International Journal of Women's Dermatology* (IJWD), everyone agreed that making our reach as inclusive and international as possible was critically important. The WDS has members from five continents and, to that end, both the *IJWD*’s editorial board and advisory board also have international representation across the globe, including Argentina, Australia, Brazil, Denmark, France, Iran, India, Israel, Germany, Greece, the Philippines, and the United States. What does *international* actually mean? According to the Oxford English dictionary, international means “across several countries”; hence, the inclusion of articles from North America among articles from South America, Europe, the Middle East, Asia, and Oceania does not mean that articles contributed from the United States are not international. Otherwise, the term *international* would be misrepresented in a U.S.-centric view of the world.

Within the IJWD, our focus is not just on the skin and hair disease of women and their families, but also on issues faced by women in the field of dermatology in their careers. Dermatology is one of the most competitive fields of medicine to gain entry into and requires top grades in medical school, research and publications, and connections to go beyond just training and into a successful academic, public hospital, or private practice career, along with hopefully a good personal life. The IJWD's January 2020 issue focused on the gender gap in academic dermatology and the supporting the personal and professional lives of female dermatologists. The IJWD March 2021 issue focused on skin of color and diversity and inclusion initiatives. Both issues highlighted many of these aspects for patients and dermatologists alike (Murase and Murrell, 2021; Shinohara, 2019).

Another way that the IJWD and WDS foster the careers of women in dermatology is to enable them to submit and publish interesting case letters, research letters, clinical pearls, editorials, review articles, and original research to our journal. In this issue, our signature piece, the Women's Health Highlight, comes from India regarding an issue very important to women: hirsutism (Mahajan, 2021). We also include a potpourri of international articles from Iran (Firooz, 2021), Denmark (Johansen, 2021), Nigeria (Anaba, 2021), Canada (Shelley, 2021), Argentina (Dickson, 2021), and the United States. The International Sections Committee of the WDS was created to increase our understanding and appreciation of the role of female and male dermatologists in all countries and to foster networking and an exchange of ideas among dermatologists at an international level, with members from Albania, Bulgaria, Canada, Israel, Mexico, Sri Lanka, the United Kingdom, and the United States (WDS, 2021a). The WDS has a mentorship program for dermatology residents in North America and Mexico, which can include international travel, so long as a mentee or mentor is female, to foster international collaboration and mentorship (WDS, 2021b). Recipients have developed relationships with mentors in Europe, Asia, Africa, North America, South America, and Australia. In addition, the WDS sponsors travel awards for female board-certified (or board-eligible) dermatologists from outside the United States and Canada to attend the WDS Annual Meeting Luncheon and the American Academy of Dermatology Annual Meeting (WDS, 2021c).

This past week on May 5, 2021, the field of dermatology lost an important woman who had enabled many of the career goals of women in dermatology throughout the world: Cynthia (Cindy) Froehlich (International Society of Dermatology [ISD], 2021a). For more than 3 decades, Cindy was the executive director of the ISD, an organization that focuses on promoting international exchange and education in dermatology across the globe and publishes the *International Journal of Dermatology*, whose editor is also an Egyptian woman, Dr. Rokea el Azhary, from the Mayo Clinic. Cindy was born in Princeton, New Jersey, and was a Fulbright scholar in French-speaking Guinea, Africa. There, she met her future husband, who also became a Fulbright scholar in the United States. Cindy ran the ISD almost single handedly for many years. She died tragically in a car accident in Maryland (Dignity Memorial, 2021).

The ISD's mentorship program, currently co-chaired by IJWD editorial board members Prof. Rashmi Sarkar from India and Dr. Martin Kassir from Dallas, has supported both female and male dermatologists from poor countries to learn from experts in other countries for periods of up to 3 months (ISD, 2021b). Cindy was the lynchpin of this program. The ISD supports approximately 10 such mentorships annually, which have been curtailed during the COVID-19 pandemic. The ISD will also be supporting 60 registration scholarships for dermatologists from poorer countries to attend its next quadrennial world congress, which Dr. Dedee Murrell is presiding over in Australia (November 10-13, 2021; now to be a virtual international congress; ISD, 2021b). In addition, the Maria Duran awards recognize women in dermatology with fellowships, a lectureship, and a medal (ISD, 2021c). As a direct result of Cindy's sudden death, there has been an outpouring of feeling from dermatologists across the globe who interacted with her (ISD, 2021a). She embodied the spirit of international collaboration and the support of women in dermatology that our journal and society value and support with our own initiatives.
